# A 5‑year update of patients with HPV positive versus negative oropharyngeal cancer after radiochemotherapy in Austria

**DOI:** 10.1007/s00508-017-1171-5

**Published:** 2017-02-15

**Authors:** Claudia Lill, Barbara Bachtiary, Edgar Selzer, Martina Mittlboeck, Dietmar Thurnher

**Affiliations:** 1 Department of Otorhinolaryngology, Medical University of Vienna, Waehringer Guertel 18–20, 1090 Vienna, Austria; 2 Rinecker Protone Therapy Center, Munich, Germany; 3 Department of Radiotherapy, Medical University of Vienna, Vienna, Austria; 4 Center for Medical Statistic, Informatics, and Intelligent Systems, Medical University of Vienna, Vienna, Austria; 5 Department of Otorhinolaryngology, Medical University of Graz, Graz, Austria

**Keywords:** Oropharyngeal cancer, Human papilloma virus, Radiochemotherapy, Update

## Abstract

**Background:**

After publishing promising results for the treatment of patients with human papilloma virus (HPV) positive oropharyngeal cancer with radiochemotherapy regarding 2‑year survival, we present an update of the disease-specific and disease-free survival after 5 years.

**Patients and methods:**

A total of 29 patients of which 18 were HPV negative and 11 HPV positive with squamous cell carcinoma of the oropharynx received radiation therapy with or without chemotherapy (cisplatin) or immunotherapy (cetuximab) between 2007 and 2009. At time of the present analysis, six patients are still alive including four with HPV positive and two with HPV negative oropharyngeal carcinoma, while 15 out of 16 patients with HPV negative tumors died and 1 died of another cause with evidence of disease.

**Results:**

Since the 2‑year disease-specific survival of patients with HPV positive cancer of the oropharynx was published with 100% versus 30.4% in HPV negative tumors, we now present the 5‑year disease-specific survival after treatment, which was 85.7% in HPV positive versus 11.1% in HPV negative patients.

**Conclusion:**

We present the results of patients receiving radiochemo(immuno)therapy for oropharyngeal cancer regarding the HPV status, which is still promising.

## Introduction

The development of squamous cell carcinomas of the head and neck (SCCHN) is mainly caused by commonly known risk factors including alcohol and tobacco abuse. In the last 15 years human papilloma virus (HPV) infections became recognized as a major risk factor for oropharyngeal cancer [1–3]. The prevalence of SCCHN with over 500,000 new cases per year worldwide is high [4], whereas the incidence of pharyngeal carcinoma is approximately 136,000 and oropharyngeal cancer is on the rise [5]. In Austria, the incidence of head and neck cancer increased from 567 newly diagnosed carcinomas in 1983 to 1085 head and neck cancer patients in 2012, while 418 patients with head and neck carcinomas died related to the disease in 1983 and 493 patients in 2014 [6]. A marked increase in the incidence of oropharyngeal cancer has been observed in the last decade in Austria [6] and other countries [7, 8]. At the same time HPV positivity increased 2.9 fold in the USA [7] and in Europe [8]. The incidence of oropharyngeal carcinoma predominantly increased in younger patients and in this patient collective especially in male patients, probably because of the higher prevalence of HPV in cervical than in penile tissue [9, 10], transmitted by orally performed sexual contact [10]. Recently, HPV detection in biopsies of head and neck cancer taken during routinely performed panendoscopy became routine practice. On average, 23.5% of oral cavity cancers, 24% laryngeal, 36.5% oropharyngeal and approximately 50% of tonsil carcinomas are HPV positive [1, 9] and we and others experienced that patients with HPV positive oropharyngeal cancer show a better outcome concerning response to treatment, overall and disease-free survival.

At present, no recommendations for modification of therapy exist, but there are de-escalation studies, e. g. replacing cisplatin by cetuximab in HPV positive patients [11, 12]. Another study consists of an induction chemotherapy with docetaxel, cisplatin and 5‑FU. In the case of a response, a reduced dose of radiotherapy plus/minus chemotherapy is administered, in the case of stable or progressive disease standard radiochemotherapy is performed [13, 14].

In the present manuscript we present the current results of disease-specific overall survival and disease-free survival of HPV positive versus negative patients with cancer of the oropharynx treated with radiochemotherapy or radioimmunotherapy in the years 2007–2009.

## Patients and methods

The study presented in 2010 was performed as a retrospective analysis [9]. All patients with oropharyngeal cancer who refused surgical treatment or where the tumors were locally unresectable (T4b, non-resectable according to the UICC/AJCC classification [9]) were included in the study. For this analysis, 88 patients with SCCHN were screened for HPV during routinely performed panendoscopy between 2007 and 2009. A HPV multiplex PCR (polymerase chain reaction) and an anti-HPV in situ hybridization as well as a routinely accomplished histological examination were carried out earlier [9]. Of these 88 patients, 45 had their tumor localization in the oropharynx, of these 30 underwent conservative treatment comprising radiotherapy, radiochemotherapy or radioimmunotherapy. This patient collective consisted of 18 HPV negative and 12 HPV positive patients. One patient was excluded from the study due to a severe HIV infection. The detection of HPV by PCR and in situ hybridization was previously published elsewhere [9].

### Radiotherapy, chemotherapy and immunotherapy

The regimen was published previously and therapy was decided in an interdisciplinary tumor board with respect to the patients request, performance status and comorbidities. The radiation therapy was administered as a standard fractionation at 2 Gy/fraction/day for 5 days per week to 70 Gy/35 fractions/7 weeks or with accelerated fractionation with concomitant boost at 1.8 Gy/fraction/day, 5 days/week and 1.5 Gy/fraction/day to a boost field as a second daily treatment for the last 12 therapy days to 72 Gy/42 fractions/6 weeks [9].

In the case of radiochemotherapy, patients received cisplatin in a dosage of 100 mg/m^2^ every 3 weeks for the duration of radiotherapy to a cumulative dose of 300 mg/m^2^. Patients who were not eligible for chemotherapy (reduced performance status, elderly patients >70 years, impaired renal function) received immunotherapy consisting of cetuximab 400 mg/m^2^ initial dose 1 week prior to irradiation and a weekly dose of 250 mg/m^2^ for 7 weeks. In three cases, patients received radiotherapy alone because of reduced performance status, age and impaired renal function. One patient was not treated at all for cancer as treatment was refused and the patient died 3 months after the initial diagnosis [9].

### Statistical analysis

Statistical evaluation was performed as previously described [9] but for convenience, the description will be reprinted. Kaplan-Meier curves represent the survival data and differences between the curves are tested with log-rank test. All deaths, independent of their cause, were considered as events for overall survival. The disease-specific survival describes only deaths concerning the underlying disease and observations with deaths unrelated to disease were censored at the time of death. For disease-free survival all recurrences of the carcinoma were considered as events [9]. The disease-specific overall survival delineates the main outcome parameter, as second outcome parameter we considered disease-free overall survival. All *p*-values are two-sided and *p* ≤ 0.05 was considered significant. All calculations were performed with SPSS (PASW Statistics, New York, NY. Version 21.0.0.0).

## Results

In this study 30 patients with oropharyngeal cancer including carcinomas of the tonsils and base of the tongue were treated with radiation, radiochemotherapy or radioimmunotherapy in the years 2007–2009 and were retrospectively evaluated. One patient presenting with a T4 HPV positive carcinoma of the tonsils was excluded from evaluation because of a severe HIV infection and subsequent complete change of the therapeutic regimen. The median age at time of therapy was 62 years (range 46–89 years) [9]. The patient collective included 19 male and 10 female patients, of these 6 patients are still alive and 23 have died. Of the patients who died 7 were HPV positive and 16 HPV negative [9]. Among the HPV positive patients, one patient died due to carcinoma, while six died of other causes including pneumonia, myocardial infarction, dilated cardiomyopathy and apoplectic stroke (Table 1). Of the patients with HPV negative carcinoma of the oropharynx, 15 died of disease and 1 died because of a colon perforation while presenting evident oropharyngeal carcinoma formations.

**Table 1.** Causes of deaths in HPV positive versus negative patients

	HPV+	HPV−
Mean age (years, range)	62 (49–89)	62 (46–82)
Male/female	9/2	10/8
*Cause of death*		
Apoplexy	1	–
Myocardial infarction	2	–
General condition	1	–
Pneumonia	1	–
Cardiomyopathy	1	–
Colon perforation	–	1
Tumor-related death	1	15
Alive and well	4	2

**Fig. 1:**
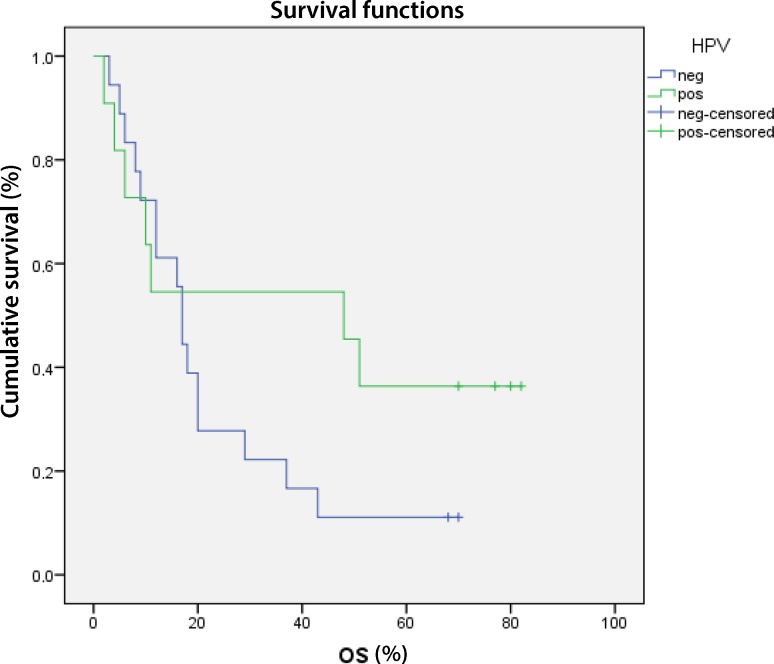
Kaplan-Meier curve of the overall survival (OS) of all patients. Subsequently, the deaths not related to cancer were censored (Fig. 2). *Blue line* survival of HPV positive patients, *green line* HPV negative

**Fig. 2:**
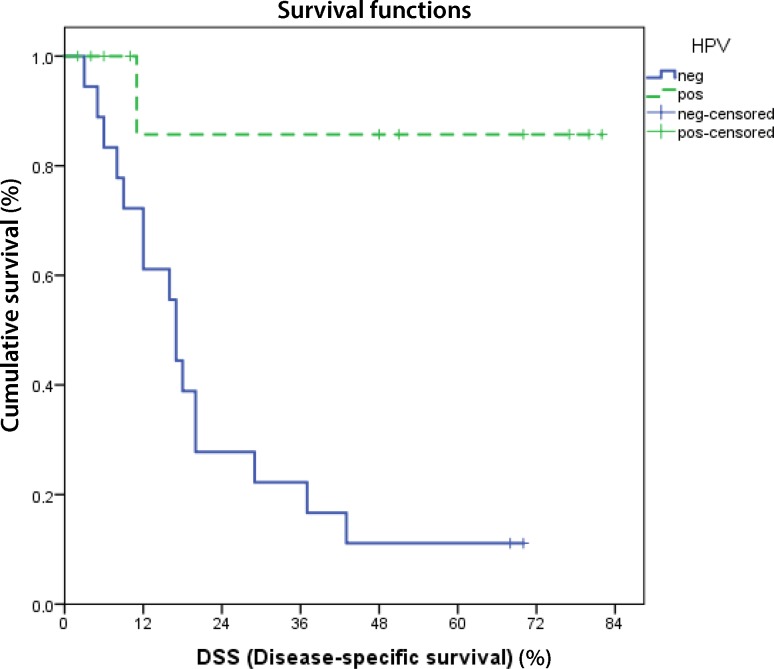
Kaplan-Meier curve of disease-specific survival (DSS). Disease-specific survival of patients with HPV positive oropharyngeal cancer is significantly enhanced after 5 years (*p* = 0.002). Non-disease-related deaths were censored. *Solid line* survival of HPV positive patients, *dashed line* HPV negative

All patients received radiation in combination with immunotherapy (cetuximab), chemotherapy (cisplatin) or radiation alone [9]. Regarding tumor stage of patients, 6 patients presented with T2 stage, 2 with T3 and 21 with T4, while HPV positive patients collective comprised 6 with T2, 1 with T3 and 4 with T4 and HPV negative included 1 with T3 and 17 with T4 tumors [9]. After a period of 5 years the overall survival of all patients was not significantly enhanced in the HPV positive group (*p* = 0.190) due to a high rate of non-disease-related deaths (*n* = 6) (Fig. 1). After censoring the patients who died of other causes [9], the disease-specific overall survival was calculated. The disease-specific overall survival of patients with HPV positive oropharyngeal carcinoma was significantly better than in patients with HPV negative tumor (*P* = 0.002) (Fig. 2).

**Fig. 3:**
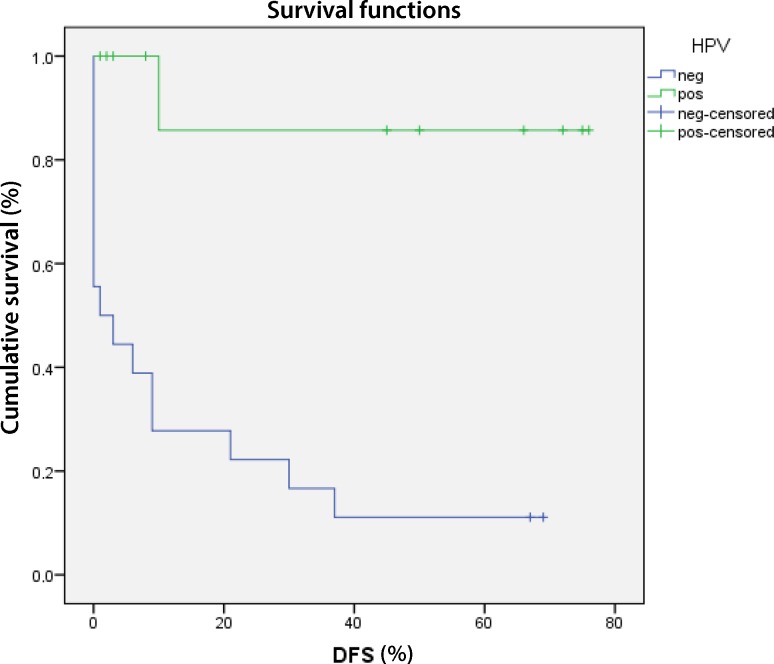
Kaplan-Meier curve of the disease-free survival (DFS) was highly significant in patients with HPV positive oropharyngeal carcinoma (*p* < 0.001). *Blue line* survival of HPV positive patients, *green line* HPV negative

The time to first event, recurrence of death of any cause was significantly improved in patients with HPV positive oropharyngeal cancer (*p* = 0.042). After censoring non disease-related deaths of patients, the disease-free survival was highly significant (*p* < 0.001) (Fig. 3).

**Fig. 4:**
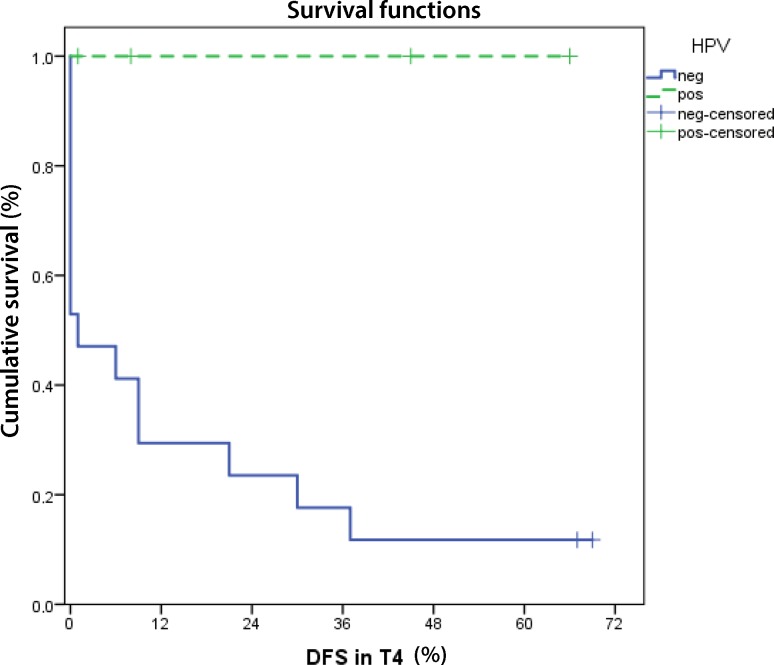
Kaplan-Meier curve of disease-free survival in patients presenting T4 tumor (DFS in T4). DFS is enhanced in HPV positive patients (*p* = 0.016). *Solid line* survival of HPV positive patients, *dashed line* HPV negative

Considering only patients with T4 tumors, they are subdivided into 4 patients with and 17 without HPV positivity. Significant differences could be observed for both disease-specific overall survival of T4 tumors (*p* = 0.042) as well as disease-free survival (*p* = 0.016) (Fig. 4).

The 5‑year disease-specific overall survival was 85.7% in patients with HPV positive versus 11.1% in HPV negative tumors.

## Discussion

We intended to perform an update of the promising results from 2010, 5 years after therapy of patients for HPV positive and negative oropharyngeal carcinomas. In our manuscript presented in 2010 [9] we could describe a 1-year disease-specific survival of HPV positive patients comprising 100% versus HPV negative patients with 72.9%, whereas the 2‑year overall survival was 100% compared to 30.4%. Both results were highly significant, at the time presenting a *p*-value of *p* = 0.015.

The present evaluation was based on the same patient collective and a re-evaluation was performed 5 years after patients started radiotherapy with or without chemotherapy or immunotherapy for oropharyngeal cancer. All patients were tested for HPV. Only the 29 patients evaluated in the first publication were updated at the present study, which surely limits the conclusion of our analysis as we present a small group of patients.

Of the patients evaluated, six patients are still alive, two of them are HPV negative and four HPV positive. Of the patients 23 have died with 7 patients being HPV positive and 16 HPV negative. The patients with HPV positive tumor died predominantly (6 out of 7) of other causes including apoplectic stroke, myocardial infarction, pneumonia and reduced performance status in a 87-year-old woman as mentioned before and listed in Table 1. The deaths were not documented as therapy-related deaths and none of these patients had evident disease. Only one patient with an HPV positive oropharyngeal carcinoma experienced a regional recurrence 10 months after therapy and died because of recurrence one month later. On the other hand, 15 of 16 patients with HPV negative tumor died because of a progression of their cancer, including one patient who died of a colon ulceration but with an evident oropharyngeal local recurrence. Of the patients with HPV negative tumor 9 showed progression of the disease even during therapy, 1 patient had a recurrence after 3 months, 4 after 9 months, 1 after 11 months and 3 patients had late recurrences after 21, 37 and 67 months, respectively. As the mean age was equivalent in both groups, the high rate of non-disease-related deaths cannot be ascribed to a higher age or more comorbidities in one group.

Overall and disease-free survival were significantly better even matching T4 HPV positive and negative patients with the limitation that merely four patients with HPV positive cancer were included to the analysis. These four patients had a T4a tumor with (N2b-c) and without (N0) lymph node metastases. In a recently implemented new staging system for HPV positive oropharyngeal cancer patients, they fulfil criteria for new stage III, which is defined by T4a-b N0-3 [15].

Concerning the outcome, we could not find differences in patients receiving immunotherapy versus chemotherapy. In the HPV positive patient group, seven patients received radioimmunotherapy, two patients each radiotherapy alone or radiochemotherapy. The patients presenting with HPV negative tumors were treated with radioimmunotherapy in seven cases, radiochemotherapy in nine and radiotherapy in one case. One remaining patient refused therapy at all. Interestingly, radiochemotherapy was administered more often in the HPV negative group in our patient collective. Nevertheless, it was a retrospective analysis and at the time of therapy no recommendations for de-escalation of therapy existed.

It has been sufficiently evidenced that patients presenting HPV positive oropharyngeal cancer show improved response to treatment and have a better disease-specific as well as disease-free survival [9, 16–19]. The 5‑year overall survival has been specified with 83% in HPV positive versus 37% [20], which is comparable to our results, predominantly concerning the HPV positive patients. As described, the 5‑year disease-specific survival was 85.7% in HPV positive versus 11.1% in HPV negative cancer patients in our study. The 3‑year disease-free survival is similar with 85.7% versus 22.2%, respectively. Another paper observed that 90% of their patients with oropharyngeal carcinoma were HPV positive and could show a 3-year OS of 93% and a 5‑year OS of 89% [21]. Not only the prediction of treatment response, but also the survival prognosis of patients with HPV positive oropharynx carcinoma is improved, which is additionally reflected in the minimized risk of second primaries, stated with 5.6% in HPV positive versus 14.6% in HPV negative cancer patients [22]. Consequentially, a reduction of therapeutic agents has to be discussed and this discussion has already started with a presentation at the American Society of Clinical Oncology (ASCO) meeting in 2012. Two studies with an overall reduced irradiation dose and replacement of cisplatin by cetuximab in patients with HPV positive oropharyngeal cancer have been presented [14]. One study by Mehrotra et al. reduced the dose of radiotherapy (±chemotherapy) to 66 Gy in the case of complete or partial response to an induction chemotherapy consisting of docetaxel, cisplatin and 5‑floururacil. In the group without response to treatment (stable disease or progression) patients received a standard radiochemotherapy [23]. A second study (ECOG 1308) with induction chemotherapy (paclitaxel, cisplatin, cetuximab) was followed by radiation therapy with a dose of 54 Gy and cetuximab only in the case of a complete response after three cycles of induction chemotherapy [13]. Patients with partial response or stable disease were conventionally treated with standard radiotherapy and cetuximab [13].

These two studies are the first attempts to enhance patient quality of life in a distinct group of HPV positive oropharyngeal cancer patients. As this group has customarily less common risk factors, such as tobacco and alcohol consumption, the prognosis is fairly good. A reduced dose of radiotherapy and chemotherapeutic agents and an extended application of the monoclonal Epidermal Growth Factor Receptor (EGFR) antagonist cetuximab with a lower potential of adverse events might improve the quality of life of these cancer patients without the risk of an early recurrence. The ECOG 3311 is a phase II trial de-escalating adjuvant radiotherapy (50 Gy compared to 60 Gy) in the case of intermediate risk including patients with less than 1 mm extracapsular extension and 2–4 positive lymph node metastases or close margins [24]. So far, there are no worldwide recommendations for reduction of therapy agents and we need to anticipate the results of the de-escalation trials to adjust the therapy for oropharyngeal cancer patients [26].

In addition, the potential to prevent HPV related tumors, be it cervical or oropharyngeal cancer, is implicated by HPV vaccination, which is included in school vaccination programs at no charge in Austria since February 2014. Currently, a 9-valent vaccination is applied and achieved in 60% of girls and 40% of boys in Austria, leading to a predicted reduction of cervical cancer in 92%, anal cancer by 83% and should also impact on reduction of the occurrence of oropharyngeal cancer [25].
